# Effect of Nd:YAG Low Level Laser Therapy on Human Gingival Fibroblasts

**DOI:** 10.1155/2015/258941

**Published:** 2015-10-04

**Authors:** Andreas S. Gkogkos, Ioannis K. Karoussis, Ioannis D. Prevezanos, Kleopatra E. Marcopoulou, Kyriaki Kyriakidou, Ioannis A. Vrotsos

**Affiliations:** Department of Periodontology, School of Dentistry, University of Athens, 2 Thivon Street, Goudi, 115 27 Athens, Greece

## Abstract

*Aim*. To evaluate the effect of Low Level Laser Therapy (LLLT) on human gingival fibroblasts in terms of proliferation and growth factors' secretion (EGF, bFGF, and VEGF). *Materials and Methods*. Primary cultures of keratinized mucosa fibroblasts were irradiated by a Nd:YAG laser 1064 nm with the following energy densities: 2.6 J/cm^2^, 5.3 J/cm^2^, 7.9 J/cm^2^, and 15.8 J/cm^2^. Controls were not irradiated. Cultures were examined for cell proliferation and growth factors' secretion after 24, 48, and 72 hours. All experimental procedures were performed in duplicate. Data were analyzed by Student's *t*-test (*p* < 0.05). *Results*. All laser-irradiation doses applied promoted a higher cell proliferation at 48 hours in a dose-response relationship compared to controls. This difference reached statistical significance for the cultures receiving 15.8 J/cm^2^ (*p* = 0.03). Regarding EGF, all laser irradiation doses applied promoted a higher secretion at 48 hours in a reverse dose-response pattern compared to controls. This difference reached statistical significance for the cultures receiving 2.6 J/cm^2^ (*p* = 0.04). EGF levels at the other time points, bFGF, and VEGF showed a random variation between the groups. *Conclusion*. Within the limits of this study, LLLT (Nd:YAG) may induce gingival fibroblasts' proliferation and upregulate the secretion of EGF. Further studies are needed to confirm these results.

## 1. Introduction

Laser devices, almost 50 years after their introduction, find numerous applications in health sciences and are used successfully in several dental specialties. At a high output power, lasers cause thermomechanical ablation used for incisions and hard or soft tissue removal. However, at a low output power (0.2–0.5 W), referred to as Low Level Laser Therapy (LLLT), they may present a stimulatory effect, via a photobiologic phenomenon (photobiomodulation), promoting tissue healing, reducing inflammation, and inducing analgesia [1]. The exact biological mechanisms that explain LLLT's effect are still a matter of research.

It has been reported that irradiation with red or near-infrared light can lead to the activation of mitochondrial respiratory chain components and the initiation of a signaling cascade which promotes mitosis and growth factors' secretion [2]. Growth factors represent keystones in the wound healing procedures. Specially, blood-derived fibroblast growth factor (bFGF) has anti-inflammatory effect [3], vascular endothelial growth factor (VEGF) plays a role in angiogenesis, inflammation, and wound healing [4, 5], and epidermal growth factor (EGF) promotes a dose-dependent migratory response in gingival fibroblasts accelerating wound healing [6].

It has been shown that LLLT may influence the proliferation of various cells participating in oral wound healing process, such as gingival fibroblasts [7], gingival epithelial cells [8], periodontal ligament cells [9], osteoblasts [10], and bone mesenchymal stem cells [11]. Although several studies support the stimulating effect of LLLT on gingival fibroblasts, the vast majority of them used diode lasers [7, 12–14]. Literature data using neodymium-doped yttrium aluminum garnet (Nd:YAG) 1064 nm lasers are very rare. Additionally, the aforementioned studies present heterogeneity in terms of wavelengths, output powers, time of application, energy densities, and technical parameters such as the type of optical fiber and the distance between optical fiber and targeting cells. The determination of parameters that could optimize LLLT's impact on wound healing of periodontal tissues is very important. A positive upgrowth stimulation effect of LLLT on gingival fibroblasts could have clinical applications in both nonsurgical and surgical periodontal therapy.

Thus, the aim of this* in vitro* study was to evaluate the effect of various energy densities of LLLT, performed with a Nd:YAG laser (1064-nm) on human gingival fibroblasts in terms of cells' proliferation and specific growth factors' secretion (EGF, bFGF, and VEGF), at certain time points after irradiation.

## 2. Materials and Methods

### 2.1. Biopsy Collection

For the purpose of this* in vitro* study, connective tissue specimens were obtained by two healthy nonsmoking donors (1 female 30 ys, 1 male 30 ys). Biopsies were performed during second-stage implant surgeries at the Postgraduate Clinic, Department of Periodontology, National and Kapodistrian University of Athens. Immediately after flap elevation, 6 × 6 mm specimens were collected from the connective tissue part of the flap. All tissue collections were carried out according to the approved guidelines set by the Human Ethics Board of Kapodistrian University of Athens, School of Dentistry.

### 2.2. Cell Cultures

Specimens were carefully sliced into 3 mm slides. Explants plated on 35 mm dishes, produced outgrowths composed of fibroblasts, after culture in Dulbecco's Modified Eagle's Medium (DMEM; Gibco Grand Island, NY) supplemented with 10% bovine calf serum (Grand Island, NY), 100 U/mL penicillin G sodium, 100 mg/mL streptomycin sulfate, and 250 pg/mL amphotericin B (Gibco, Grand Island, NY). The obtained fibroblasts grew on standard conditions (37°C, 85% humidity, and 5% CO_2_). After the first passage (cells reached 80%–85% confluence), cultures were subjected to immunomagnetic cell sorting using STRO-1 (BioLegend, San Diego, CA, USA) and anti-IgM MicroBeads (Miltenyi Biotec, Bergisch Gladbach, Germany) according to the manufacturers' instructions (MACS; Miltenyi Biotec, Bergisch Gladbach, Germany), in order to determine the cells' type (fibroblasts). The experiment took place at the third passage. After cultures' growth, cells were trypsinized and seeded at 12-well multiwall (3.8 cm^2^) plates (10000 cells/mL) in DMEM 10% fetal bovine serum (FBS). Before laser irradiation the medium of samples and controls was completely removed and replaced with serum-free DMEM.

### 2.3. Laser Irradiation

**Figure 1:**
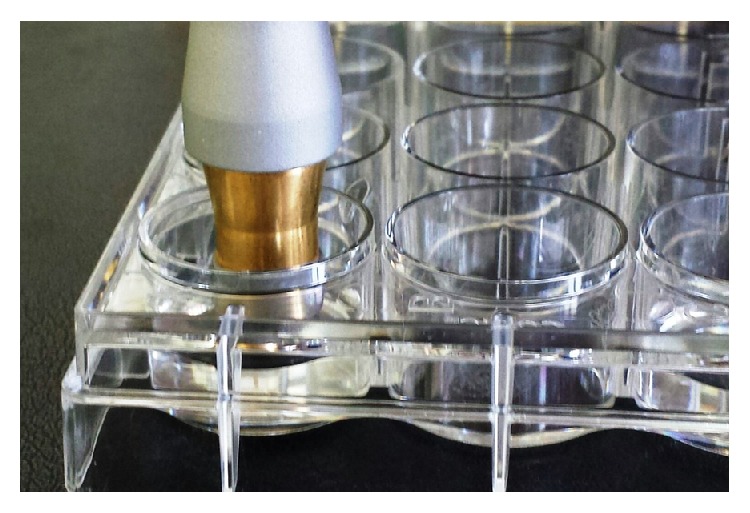
The irradiation was carried out with manipolo per terapia N4060 handpiece, approximately 5 mm above cell level.

The irradiation was performed with a Nd:YAG laser (1064-nm, DEKA Smart File) and a prefabricated, commercially available, handpiece (manipolo per terapia N40601). The laser beam was directed perpendicularly to the cell level from a distance of 5 mm. Cells were irradiated for 20, 40, 60, or 120 seconds (Figure 1).

The irradiation settings were power 0.5 W, frequency 10 Hz, energy 50 mJ, and pulse duration ≥700 *μ*sec. The corresponding energy densities were 2.6 J/cm^2^, 5.3 J/cm^2^, 7.9 J/cm^2^, and 15.8 J/cm^2^, respectively (Table 1). A wide range of energy densities was selected, to investigate all the possible effects of LLLT with Nd:YAG laser on gingival fibroblasts, either positive or negative. Control cultures were not irradiated. All experiments were performed in duplicate.

### 2.4. Proliferation Assessment

Cells were counted after trypsinization (0,05% trypsin/EDTA in PBS for 5 minutes) at day 0 (baseline levels), 24, 48, and 72 hours after irradiation for both irradiated and control groups. The proliferation was assessed with an optical method. In an inverted optical microscope (Zeis), cell counting was performed over a hemocytometer, by an experienced blinded (the examiner was not aware of the treatment for each culture) biologist.

### 2.5. Growth Factors Assay

Growth factors' secretion was assessed with Luminex technology. Luminex's xMAP technology is based on a combination of flow cytometry, microspheres, lasers, digital signal processing, and traditional chemistry. The technique involves Luminex's 100 distinct sets of tiny color-coded beads, called microspheres. Each bead set can be coated with a specific capture probe or Anti Tag to allow the capture and detection of specific targets. The technology allows rapid and precise analysis of several protein molecules, within a single reaction.

Supernatants were collected at day 0 (baseline levels), 24, 48, and 72 hours after irradiation. A quantitative analysis of EGF, bFGF, and VEGF levels was performed, according to the manufactures' recommendations (Luminex Human Growth Factor 4-plex Panel Kit, Invitrogen, CA).

### 2.6. Statistical Analysis

To investigate differences in cells' number between each test group and controls, we used* Student's t-test*. For the comparison of growth factors (EGF, VEGF, and bFGF) we first calculated the change from the baseline measurement for each group and we compared those changes using *t-test*, at every time point. All tests were two-sided at *α* = 5% level of statistical significance.

## 3. Results

**Figure 2:**
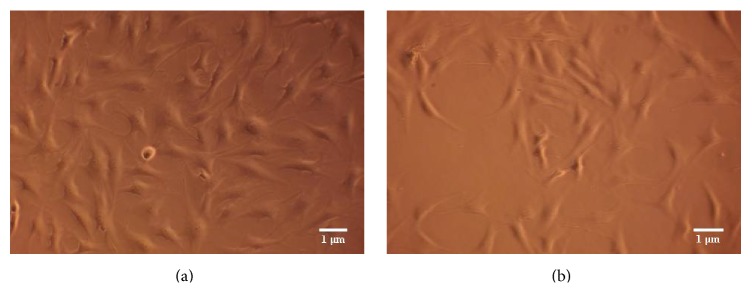
Human gingival fibroblasts at 48 hours. (a) Irradiated with Nd:YAG laser for 120 (120 s group) (original magnification, ×100). (b) Nonirradiated (Ctrl group), (original magnification, ×100).

All laser-irradiation doses applied promoted a higher cell proliferation at 48 hours compared to control group. This difference reached statistical significance for the group irradiated for 120 s versus control group (mean: 68000, SD: 6324.555, 95% CI: 57936.22–78063.78, and *p* = 0.03) (Figures 2(a) and 2(b)).

A dose-response relationship, at 48 h may be implied. At 72 hours, all laser-irradiated groups (except 20 s) cells' number was higher than controls. The growth curves are shown in Table 2 and Figure 4.

**Figure 3:**
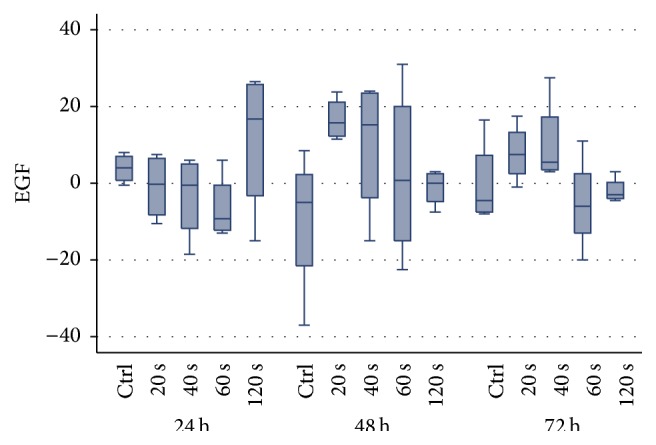
Boxplot of EGF values differences from baseline for each radiation time and control, after 24, 48, and 72 hours.

**Figure 4:**
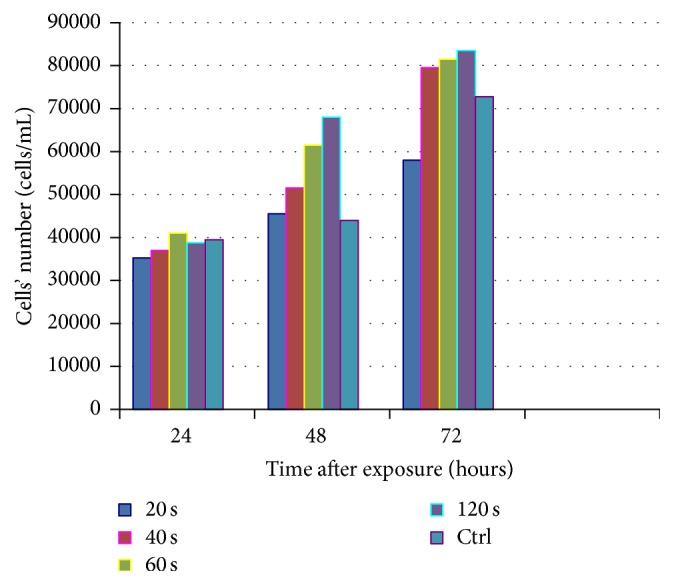
Cells' number of control and test groups 24, 48, and 72 hours after irradiation.

Growth factors' secretion results are presented in Table 3. Regarding EGF, all laser-irradiation doses applied promoted a higher secretion at 48 hours compared to control group. This difference reached statistical significance for the group irradiated for 20 s versus control group (mean: 16.7, SD: 5.608923, 95% CI: 7.774953–25.62505, and *p* = 0.04) (Figure 3). A reverse dose-response relationship, at 48 h, may be implied. As far as VEGF, at 48 h, values were higher or equal to controls. EGF and VEGF values, at the other time points (24 and 72 hours) and bFGF as well, showed a random variation between the groups.

## 4. Discussion

### 4.1. LLLT and Cells Proliferation

Within the limitations of this study (sample size and arbitrary energy densities), it appeared that treatment with low power laser (LLLT) using Nd:YAG laser, specifically in irradiation time of 120 s (energy density: 15.8 J/cm^2^), resulted in a statistically significant increase of cells' population (*p* = 0.03), compared to the control group, 48 hours after irradiation. In the international literature, some publications on the effect of laser devices on gingival fibroblasts, skin fibroblasts, and animal fibroblasts can be found [3, 15–19]. The heterogeneity, among these studies, regarding the type of laser devices used and their settings (wavelength, energy density), does not allow a direct comparison between them and exportation of a detailed conclusion. However, it is generally supported, in most of the studies, that LLLT increases the cell population of gingival fibroblasts [7, 13, 20–22]. A recent systematic review, about the effect of LLLT in various human and animal cell cultures, showed the ability of laser to modulate cellular proliferation. However, finding the most appropriate irradiation settings is still an important piece of research [23].

It is worth mentioning that there is no equivalent study in the literature, concerning the settings of Nd:YAG laser and irradiation time. However, three studies researched the effect of Nd:YAG laser on gingival fibroblasts [15–17]. Specifically, Chen et al., in 2000, noticed evidence of a decrease in the vitality of human gingival fibroblasts (cell damage zone 2.2~4.2 mm in diameter), after irradiation (1.0–3.0 W), for 10 sec. However, when defocused irradiation was applied (2 mm from the cell level), no significant decrease in cell vitality was observed. The main difference between Chen's study and ours is the irradiation power (1–3 W versus 0.5 W). It seems that these power values cause cells' death. Furthermore, Chen et al. used a 400 *μ*m diameter fiber in contact or in 2 mm distance from cell level, in contrast with the present study, where a defocused handpiece (which ensured a uniform distribution of radiation on cells across the surface of the well) from the distance of 5 mm was used. Possibly the minus spot size (increased energy density) led to cells' death [15]. Moreover, Gutknecht et al. reported cellular (L-929 fibroblast cell cultures) damage with a necrotic zone of 8.1~10.0 mm in diameter after Nd:YAG laser irradiation (2.1~3.0 W) for 30 seconds [16]. It is obvious that at these two studies examined the possible hazard effect of Nd:YAG laser irradiation on gingival fibroblasts' vitality. In our study, the aim was to investigate the possible beneficial effects of Nd:YAG laser, using low level settings.

In another* in vitro* study, in skin fibroblasts, it appeared that the application of the Nd:YAG laser and KTP laser at high doses (10–40 J/cm^2^ and 3–12 J/cm^2^, resp.) resulted in structural damage to the DNA of cells [24]. In corresponding results, Hawkins and Abrahamse using high doses of irradiation with a HeNe laser (632.8-nm) observed a deterioration of structural components of the membrane and the DNA of the cells. They showed better results in skin fibroblasts' proliferation and migration after irradiation with HeNe laser (632.8-nm), relative to the diode 830 nm and Nd:YAG (1064 nm) (irradiation with 5 J/cm^2^ on the first and fourth day) [25].

The other study, using Nd:YAG laser for LLLT (1.5 J/cm^2^), was from Chellini et al. in 2010, who showed that fibroblasts' (cell line derived from mice NIH/3T3 fibroblasts) irradiation did not alter the vitality of cells, but it did not enhance cell proliferation too, 24 and 48 hours after irradiation. Instead, LLLT resulted in significant production of type I collagen. Results indicated the possible regulator role of LLLT with Nd:YAG laser in this particular cell culture [17]. Between this study and ours are many differences (primary culture versus cell line, frequency, energy, optic fiber diameter, time, and distance of irradiation), so a direct comparison cannot be implied. However, regarding energy densities (1.5 J/cm^2^ versus 2.6 to 15.8 J/cm^2^), it can be postulated that energy densities higher than 2.6 J/cm^2^ should be used in LLLT with Nd:YAG laser to achieve gingival fibroblast proliferation.

In literature, there are studies using other laser wavelengths in LLLT. Specifically, Kreisler et al. found a statistically significant difference in gingival fibroblasts proliferation, 24 and 48 hours after irradiation with a diode laser (809 nm, 7.84 J/cm^2^) [26]. The same biostimulatory effect of LLLT in gingival fibroblasts in* in vitro* studies was found by Basso et al. in 2012 with a diode laser (780 ± 3 nm, 0.5 and 3 J/cm^2^) [13], Vinck et al. in 2003 with a diode laser (830 nm, 1 J/cm^2^) [27], Azevedo et al. in 2006 with a diode laser (660 nm, 2 J/cm^2^) [7], and Pourzarandian et al. in 2005 with a Er:YAG laser (3.37 J/cm^2^) [20].

### 4.2. LLLT and Growth Factors' Secretion

In this study, we found that all laser-irradiation doses applied promoted a higher secretion of EGF, at 48 hours, compared to control group. This difference reached statistical significance for the group irradiated for 20 s versus the control group (*p* = 0.04). A reverse dose-response relationship, at 48 h, may be implied. Potentially, this trend exists, in the other groups, like VEGF at 48 h, where all laser-irradiated group's values of VEGF were higher or equal to controls. However, due to the small sample, this claim remains to be confirmed by future studies. EGF and VEGF values at the other time points (24 and 72 hours) and bFGF as well showed a random variation between the groups.

It should be emphasized that in the literature there is no study to date to examine the secretion of EGF and VEGF from gingival fibroblasts after irradiation with Nd:YAG laser. The only relative reference is EGF secretion and LLLT, done by Mvula et al. in 2009, who studied the synergistic action of the growth factor with LLLT (636 nm, diode laser), which led to the proliferation of stem cells of adipose tissue [28].

In an experimental study on gingival fibroblast culture, it was found that irradiation with diode laser caused increased production of VEGF and TGF-b mRNA and the corresponding mRNA for the synthesis of type I collagen [29]. Also, Kipshidze et al., in 2001, showed that LLLT (He:Ne, 632 nm, 2.1 J/cm^2^) resulted in a statistically significant increase of VEGF secretion in culture of myocardium fibroblasts [30]. Dourado et al. in 2011, in an* in vitro* study in endothelial and nonendothelial cells of mice gastrocnemius, showed that LLLT with HeNe (632.8 nm) or GaAs (904 nm) laser increased angiogenesis and the proliferation of regenerating cells and decreased polymorphonuclear neutrophils [31]. Furthermore, in an* in vivo* animal (rat) study, it was found that LLLT with diode laser radiation in two different lengths (with energy density of 35 and 5 J/cm^2^, resp.) resulted in a statistically significant reduction of expression of mRNA transcripting VEGF, after injury of the rats' tongue [32]. These data suggest that LLLT accelerates wound healing.

In the present study, no difference was observed in the secretion of bFGF between groups. Safavi et al. in 2008 led to similar results while LLLT with HeNe laser (7.51 J/cm^2^) in mice gingiva showed a statistically significant increase in secretion of PDGF and TGF-b genes, 30 minutes after irradiation, but no influence to bFGF [3]. Indeed, Hawkins and Abrahamse reported that LLLT with Nd:YAG laser (1064 nm, 16 J/cm^2^) resulted in reduced secretion of TGF-b (*p* ≤ 0.05). In contrast, irradiation with HeNe laser (632.8-nm, 5 J/cm^2^) resulted in marginally significant increase in secretion of bFGF (*p* = 0.05) [25]. Moreover, Saygun et al. found a statistical significance (*p* ≤ 0.01) in bFGF expression after irradiation with a diode laser (685 nm for 140 s, 2 J/cm^2^) [21]. Damante et al. concluded similar results in an* in vitro* study with a diode laser (660 nm, 3 J/cm^2^ and 5 J/cm^2^) [14]. Finally, it has been showed that LLLT with KTP laser (532 nm, 0.8 J/cm^2^) in human skin fibroblasts led to statistically significant increase of bFGF gene expression [18]. These studies show that LLLT with diode laser, KTP, or HeNe laser possibly have an advantage compared to Nd:YAG laser, concerning bFGF secretion. On the other hand, Usumez et al. in an animal study found that Nd:YAG and 980 nm diode laser therapy (8 J/cm^2^) accelerated the wound healing process by changing the expression of PDGF and bFGF [33].

Finally it is transpired that LLLT with Nd:YAG laser, at the settings used in this study (energy densities 2.6 to 15.8 J/cm^2^), promoted both keratinized mucosa fibroblasts' proliferation and EGF secretion, 48 hours after irradiation. It could be postulated that a repetition of LLLT (with Nd:YAG laser) every 48 hours could possibly induce growth factors' secretion and cells proliferation after each irradiation.

## 5. Conclusion

Within the limitations of this experimental study (sample size and arbitrary energy densities), the results indicated that LLLT (Nd:YAG 1064 laser) did not cause cell death for the settings used. It appears that these settings of Nd:YAG laser are safe. Moreover, the cell proliferation of primary cultured gingival fibroblasts increased after laser irradiation, presenting a potentially dose-dependent action. LLLT (Nd:YAG, 1064 laser) contributes probably to the secretion of EGF in a reverse dose-response pattern. Finally, it becomes clear that more studies with larger sample sizes are needed, in order to draw solid conclusions. Future studies should consider evaluating growth factors, irradiation parameters, and/or laser wavelengths.

## Figures and Tables

**Table 1:** Laser parameters used during irradiation.

Laser	Nd:YAG			
Wavelength	1064 nm			
Spectrum	Near-infrared			
Irradiation mode	Pulsed wave, long pulse (≥700 *μ*sec)			
Settings	Power 0,5 W, frequency 10 Hz, and energy 50 mJ			
Duration	20 s	40 s	60 s	120 s
Energy density	2.6 J/cm^2^	5.3 J/cm^2^	7.9 J/cm^2^	15.8 J/cm^2^
Irradiation	Single treatment at day 0			
Spot area	0,785 cm^2^ (manipolo per terapia N40601)			
Distance of irradiation	5 mm			

**Table 2:** Cell counts of control and test groups for the different time points.

Cells	*N*	Mean (SD)	Median (range)	*p* value^**∗**^
24 hours				
Controls	4	39500 (10116)	41000 (28000, 48000)	
20 s	4	35250 (8382)	37000 (24000, 43000)	0.54
40 s	4	37000 (11605)	38000 (22000, 50000)	0.76
60 s	4	41000 (13216)	40000 (26000, 58000)	0.86
120 s	4	38750 (8539)	37500 (30000, 50000)	0.91

48 hours				
Controls	4	44000 (15144)	38000 (34000, 66000)	
20 s	4	45500 (5972)	44000 (40000, 54000)	0.86
40 s	4	51500 (9849)	51000 (40000, 64000)	0.43
60 s	4	61500 (5972)	60000 (56000, 70000)	0.07
120 s	4	68000 (6325)	69000 (60000, 74000)	**0.03**

72 hours				
Controls	4	72750 (10500)	69500 (64000, 88000)	
20 s	4	58000 (17205)	57000 (40000, 78000)	0.19
40 s	4	79500 (22531)	81000 (52000, 104000)	0.60
60 s	4	81500 (13796)	83000 (64000, 96000)	0.35
120 s	4	83500 (17388)	86000 (60000, 102000)	0.33

^*∗*^Versus control.

**Table 3:** Mean (SD) change from the baseline measurement for each group at every time point.

	EGF			VEGF			bFGF		
	*N*	Mean (SD)	*p* value^*∗*^	*N*	Mean (SD)	*p* value^*∗*^	*N*	Mean (SD)	*p* value^*∗*^
24 h									
Ctrl	4	3.9 (3.8)		4	2.9 (2.1)		4	−0.8 (3.6)	
20 s	4	−0.9 (8.8)	0.36	4	1.3 (0.3)	0.18	4	−2.9 (3.1)	0.41
40 s	4	−3.4 (11.2)	0.27	4	1.6 (1.3)	0.34	4	−1.9 (3.1)	0.65
60 s	4	−6.4 (8.6)	0.07	4	1.5 (0.4)	0.25	4	−0.4 (2.4)	0.87
120 s	4	11.3 (19.3)	0.48	4	4.9 (1.5)	0.18	4	−0.4 (1.8)	0.86

48 h									
Ctrl	4	−9.6 (19.3)		4	3.3 (3.0)		4	−0.4 (1.9)	
20 s	4	16.7 (5.6)	**0.04**	4	7.0 (4.2)	0.20	4	1.3 (2.4)	0.33
40 s	4	9.9 (18.2)	0.19	4	4.0 (3.8)	0.77	4	−0.6 (1.4)	0.84
60 s	4	2.5 (22.9)	0.45	4	3.3 (2.2)	0.51	4	−0.3 (2.7)	0.94
120 s	4	−1.1 (4.8)	0.43	4	6.0 (7.4)	0.42	4	0.4 (1.1)	0.52

72 h									
Ctrl	4	−0.1 (11.4)		4	3.8 (6.1)		4	0.4 (3.4)	
20 s	4	7.9 (7.7)	0.29	4	4.5 (4.4)	0.85	4	1.1 (2.3)	0.73
40 s	4	10.4 (11.5)	0.24	4	5.3 (5.7)	0.73	4	1.0 (1.1)	0.74
60 s	4	−5.3 (12.7)	0.57	4	2.6 (2.9)	0.75	4	−2.1 (3.3)	0.34
120 s	4	−1.9 (3.4)	0.78	4	3.6 (4.6)	0.98	4	−0.6 (2.3)	0.65

^*∗*^ *t*-test versus control.
